# The role of specific biomarkers, as predictors of post-operative complications following flexible ureterorenoscopy (FURS), for the treatment of kidney stones: a single-centre observational clinical pilot-study in 37 patients

**DOI:** 10.1186/s12894-020-00693-4

**Published:** 2020-08-14

**Authors:** Stephen Fôn Hughes, Alyson Jayne Moyes, Rebecca May Lamb, Peter Ella-tongwiis, Christopher Bell, Ahmed Moussa, Iqbal Shergill

**Affiliations:** 1 North Wales & North West Urological Research Centre, Betsi Cadwaladr University Health Board (BCUHB) Wrexham Maelor Hospital, Wrexham, Wales, UK; 2 North Wales Clinical Research Centre, Betsi Cadwaladr University Health Board (BCUHB) Wrexham Maelor Hospital, Wrexham, Wales, UK; 3 School of Medical Sciences, Bangor University, Bangor, Wales, UK; 4 Department of Biological Sciences, University of Chester, Chester, UK; 5 The Alan de Bolla Department of Urology, BCUHB Wrexham Maelor Hospital, Wrexham, Wales, UK

**Keywords:** Flexible Ureterenoscopy, (FURS), Kidney stones, Biomarkers, Infection, Post-operative complications

## Abstract

**Background:**

The number of patients diagnosed and subsequently treated for kidney stones is increasing, and as such the number of post-operative complications is likely to increase. At present, little is known about the role of specific biomarkers, following flexible ureterorenoscopy (FURS) for the surgical treatment of kidney stones. The main aim of the study was to evaluate the role of kidney and infection biomarkers, in patients undergoing FURS.

**Methods:**

Included were 37 patients (24 males, 13 females), who underwent elective FURS, for the treatment of kidney stones. Venous blood samples were collected from each patient: pre-operatively, and at 30 min, 2 and 4 h post-operatively. Changes to kidney (NGAL, Cystatin-C) and infection (MPO, PCT) biomarkers was quantified by means of ELISA, Biomerieux mini-vidas and Konelab 20 analysers.

**Results:**

Four patients developed post-operative complications (3 - UTIs with urinary retention, 1 - urosepsis. NGAL concentration increased significantly following FURS (*p* = 0.034). Although no significant changes were seen in Cystatin C, MPO and PCT (*p* ≥ 0.05) some key clinical observation were noted. Limiting factors for this study were the small number of patients recruited and restriction in blood sampling beyond 4 h.

**Conclusions:**

Although not confirmative, changes seen to biomarkers such as Cystatin C, NGAL and MPO in our observational clinical pilot-study may warrant further investigation, involving larger cohorts, to fully understand the role of these biomarkers and their potential association with post-operative complications which can develop following FURS.

## Background

Flexible ureterorenoscopy (FURS) is an increasingly popular procedure for the treatment of kidney stones. The complication rate following FURS is up to 25% with this figure likely to increase [1]. Conventionally in the UK, patients have been monitored post-operatively using routine blood tests, such as the full blood count, urea and electrolyte and C reactive protein (CRP). Although these traditional blood tests are clinically useful, they are non-specific in nature, and fail to identify patients at increased risk of developing postoperative complications, such as infection and acute kidney injury.

At present, there is limited literature and evidence-based reports highlighting the role of specific biomarkers, which can monitor patients following FURS. In turn, it is anticipated, in the future, that a specific panel of biomarkers (or biomarker profile) may help aid clinicians in the monitoring of patients undergoing FURS and could help identify or predict those individuals at increased risk of developing complications post-procedure.

In the present study, we have identified specific kidney and potential infection biomarkers that may provide clinically important information that will aid monitoring and subsequent managing of these patients. Cystatin C is a cysteine proteinase inhibitor which is produced by all nucleated cells and is has gathered increasing interest as a specific marker of kidney function [2].

Due to its low molecular weight (13.3 kDa), Cystatin C is freely filtered at the glomerulus, and is neither secreted nor reabsorbed in the renal tubules [3]. Although Cystatin C has been shown to be a useful biomarker for identifying renal injury post-surgery in a critical care setting [4], at present there are no studies that have reported the role of the parameter in patients undergoing FURS. This makes cystatin C a novel and exiting prospect for the monitoring of kidney stone patients.

Neutrophil gelatinase-associated lipocalin (NGAL) belongs to the lipocalin family, predominantly found within neutrophils, but are also found in low levels in the kidney, prostate and respiratory tract [5]. NGAL is reported to be a modulator of the inflammatory response, binding and attacking small lipophilic substances in bacteria such as lipopolysaccharides and formyl peptides [6]. Several studies have suggested NGAL may be a useful kidney injury biomarker, particularly in patients with nephrolithiasis or its associated pathologies [7–10]. NGAL has been reported as a potential renal injury biomarker in the early period after surgery and has a multifaceted role in inflammation and cancer [2, 11]. Notably, in their study of urinary biomarkers in 30 patients post-FURS, Benli et al (2017) reported a significant increase in NGAL levels 2 h post-operatively [2]. This suggests that the early post-operative rise in NGAL may provide an opportunity for this biomarker to be utilised as part of monitoring patients following FURS. In the present study, we aim to expand on previous work undertaken by others and to investigate changes in NGAL amongst other biomarkers up to 4 h post-FURS.

Myeloperoxidase (MPO) is an enzyme which has been shown to be expressed within neutrophils and monocytes [12]. MPO produces hypochlorous acids (HOCl) as part of the respiratory burst process, providing a highly anti-microbial environment for bacteria and other pathogens [13, 14]. It is believed to have a role in cellular homeostasis and is associated with inflammatory disease development and progression; strong link has been established in this regard, especially in the field of cardiovascular disease (CVD) [15]. Hasmann et al. (2013) have previously reported the role of MPO activity in wound fluid, as a potential marker for infection, as it can be appreciated that this biomarker may provide a more rapid diagnosis, in comparison to traditional means of testing for infection via microbiological methods and the non-specific CRP [16]. MPO therefore warrants investigation in the present study, as a potential biomarker for infection or monitoring the respiratory burst in patients post-FURS.

Sepsis, a potential life-threatening condition can be defined as bacteriuria with systemic inflammatory response syndrome (SIRS). It can be appreciated that sepsis can be caused by an array of microorganisms such as bacteria, fungi and viruses. However, bacterial sepsis is by far clinically the most diagnosed [17]. In sepsis, pathogens enter the blood stream where they can thrive and release harmful infectious factors into the bloodstream [18]. These products can stimulate the release of mediators of sepsis from biological cells such as monocytes, macrophages, neutrophils and plasma cell precursors [19]. The body’s attempt to fight the infection can involve a systemic inflammatory response (SIRS), however, the resultant activation of inflammatory proteins can result in more extensive tissue and organ damage [20]. Procalcitonin (PCT), a calcitonin precursor, is upregulated in response to such damage [21]. Raised blood PCT levels indicate systemic bacterial infection. With urosepsis a well-known complication of FURS, monitoring PCT pre and post FURS may prove beneficial in managing these patients [20, 21].

Several groups, including ours, have reported on the effect of various urological and other surgical procedures on post-operative routine and novel blood tests [22–27], However, crucially, there are few studies into the effect of FURS on kidney and infection biomarkers. Our study aimed to evaluate the role of these biomarkers in order to better establish their post-operative levels following FURS for the treatment of nephrolithiasis.

## Methods

### Subject volunteers

Ethical approval for this study was received from the Welsh Research Ethics Service (REC) 4 committee (REC4: 12/WA/0117) and were carried out in accordance with the ethical rules of the Helsinki Declaration and Good Clinical Practice. As this is a single-centre observational clinical pilot-study, no sample size or power calculation has been performed, and therefore all the comparisons that will be considered are exploratory in nature regardless of the significance level found. Thirty-seven consecutive patients undergoing elective FURS for the treatment of kidney stones were recruited (*n* = 37). Twenty-four males and 13 females aged between 28 and 87 years (median 50 years) were recruited after receiving written informed consent [23].

### Flexible ureterorenoscopy (FURS)

FURS was performed as per local protocol at the Betsi Cadwaladr University Health Board (BCUHB) Wrexham Maelor, NHS hospital, North Wales, UK by a single consultant surgeon who is an expert in the field, having undertaken > 1000 FURS procedures. The Olympus URF-P6 fibreoptic flexible Ureterorenoscope was used in all cases. This has a working channel of 3.6Fr, working length 670 mm, outer diameter 4.9Fr and outer diameter insertion tube of 7.95Fr. Height of the irrigation fluid was set to 80cmH2O and in all cases a 10/12Fr Coloplast ureteral access sheath was used, to standardise all operations. As a result, injury to the distal tubule was unrelated to the surgical procedure itself. The indication to treat the patient with a 25 mm stone was based on a multi-disciplinary decision which was patient centred, due to significant co-morbidities and as such not suitable for standard PCNL surgery. Clinical and surgical information pertaining to the present study are presented in Table 1. Where possible, stones were sent for composition analysis, using infrared spectroscopy at the Leicester Royal Infirmary, UK [23].

**Table 1:** Clinical and surgical information

Number of stones	Patients (*n* = 37)
1	24
2	10
3	3
Median Stone Size	9.1 mm (range: 3.0 mm–25.00 mm)
Median Hounsfield Unit (HU)	1123 (range: 240–2224)
Stone location	
Upper pole	15 patients
Mid pole	8 patients
Lower pole	14 patients
Median duration of laser lithotripsy	9.54 min (range: 30 s-54.02 min)
Average energy	3232.7 J (Joule)
Mean pulse	6272.76 (number of pulses)
Median Operating time	57 min (range: 20–175 min)
Complete stone clearance	100% (*n* = 37), confirmed via imaging at 3 months KUB XR if radio-opaque stone and CT if lucent
Stent insertion	24 patients

### Blood samples

Pre-operative (baseline) venous blood samples were collected, with subsequent samples collected after FURS at 30, 120 and 240 min. All samples were collected using di-potassium ethylenediaminetetraacetic acid (EDTA) and serum separator tube (SST) vacutainers. An 83% compliance was obtained regarding blood sample collection at all time points, with the remaining 17% non-compliance being due to difficulty (i.e. poor veins) with the venesection.

### Measurement of kidney biomarkers

Serum Cystatin C levels (mg/ml) were measured employing a latex enhanced immunoturbidimetric methodology, using a Thermo Fisher KoneLab 20 automated analyser. The Cystatin C kits were supplied from Randox Laboratories (*Cat No:* CYS4004).

Serum Neutrophil gelatinase-associated lipocalin (NGAL) concentrations (ng/ml) was measured using commercially available kits via quantitative sandwich enzyme immunoassays (ELISA) and were supplied by R&D Systems (*Cat No:* DLCN20).

### Measurement of infection biomarkers

Serum Myeloperoxidase (MPO) concentrations (ng/ml) were measured using commercially available quantitative sandwich enzyme immunoassay (ELISA) kits supplied by R&D Systems (Cat No: DMYE00B).

Plasma Procalcitonin (PCT) levels (ng/ml) were measured using commercially available kits (*Cat No:* 30450) and a Biomerieux mini-VIDAS automated immunoanalyser, employing a one-step immunoassay method.

### Statistical analysis

Statistical analysis was performed using SPSS (version 26). Testing for normality was carried out, and where data was parametric, repeated measures one-way analysis of variance (ANOVA) employed, using a 5% level of significance. The Bonferroni method for pairwise comparisons between means was used for post-hoc testing.

Non-parametric data were analysed using the Friedman test. Where this reached statistical significance, subsequent tests were performed with the Wilcoxon. Statistical significance was defined when *p* ≤ 0.05.

All parametric data is presented as mean ± standard deviation (S.D), whilst non-parametric results are presented as median ± interquartile range (Iqr).

## Results

### Effect on Cystatin C concentration

**Fig. 1:**
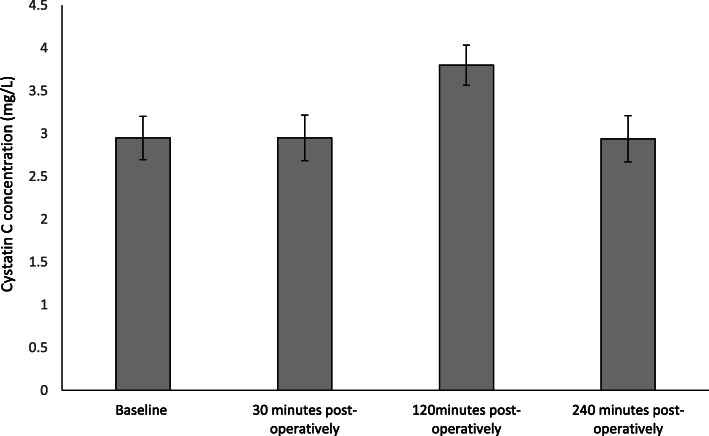
The effect of FURS, for the treatment of nephrolithiasis, on Cystatin C concentration. The points represent the median ± Iqr, *p* = 0.462 (*n* = 37)

The results are expressed as mg/ml and represent changes to serum Cystatin C following FURS (Fig. 1). Cystatin C was measured as means of a kidney biomarker. Cystatin C concentration did not change significantly from baseline (2.95 ± 2.53 mg/L) to 30 min post-FURS (2.95 ± 2.66 mg/L). At 120 min post FURS there was an increase in Cystatin C levels (3.8 ± 2.36 mg/L) followed by a decrease back toward basal levels at 240 min (2.94 ± 2.7 mg/L). There were no statistically significant changes in Cystatin C concentrations following FURS (*p* = 0.462), as determined by the Friedman test.

### Effect NGAL

**Fig. 2:**
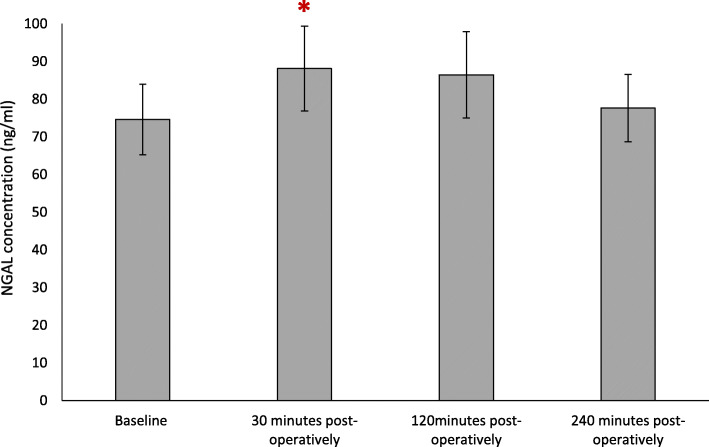
The effect of FURS, for the treatment of nephrolithiasis, on NGAL concentration. The points represent the median ± Iqr, *p* = 0.034. * = Wilcoxon test showed significance (*p* = 0.047) between levels at baseline and 30 min post-operatively *n* = 37. Normal reference range = 42-177 ng/ml

The results are expressed as ng/ml and represent changes to serum NGAL following FURS (Fig. 2). Initially, there was an increase in NGAL concentration from baseline (74.59 ± 9.4 ng/ml) up to 30 min (88.12 ± 11.25 ng/ml) post-FURS. At 120 min (86.45 ± 11.45 ng/ml) and 240 min post FURS (77.63 ± 8.90 ng/ml) NGAL concentration decreased toward basal levels. Friedman analysis illustrated a significant increase in NGAL following FURS (*p* = 0.034). Post hoc analysis employing the Wilcoxon test, confirmed a significant increase in NGAL between baseline and 30 min post-FURS (*p* = 0.047), although these changes did not correlate with surgical time (*p* > 0.05).

### Effect myeloperoxidase (MPO) concentration

**Fig. 3:**
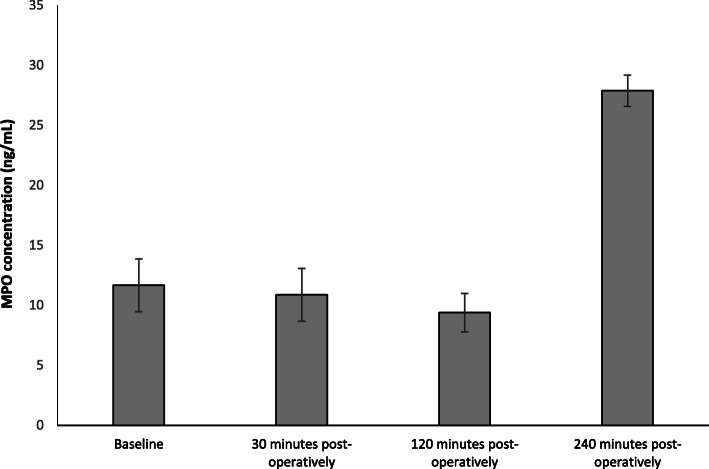
The effect of FURS for the treatment of nephrolithiasis on MPO concentration. The points represent the median ± Iqr, *p* = 0.38 (*n* = 37)

The results are expressed as ng/ml and represent changes to serum MPO following FURS (Fig. 3). MPO was measured as means of a biomarker for infection. Initially, a decrease in MPO concentration from baseline (11.66 ± 2.2 ng/ml) was seen, at 30 min (10.87 ± 2.2 ng/ml) and 120 min (9.38 ± 1.6 ng/ml) post FURS. However, a sharp increase occurred at 240 min (27.88 ± 1.3 ng/ml) post FURS. However, statistical analysis found no significant changes in MPO following FURS (*p* = 0.38), as determined by the Friedman test.

### Effect on PCT concentration

**Fig. 4:**
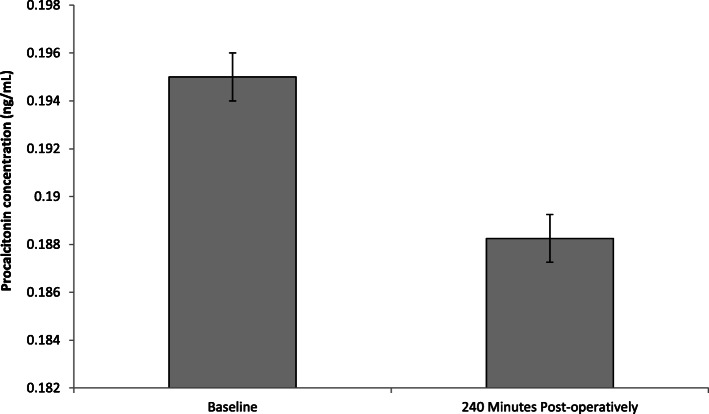
The effect of FURS, for the treatment of nephrolithiasis, on PCT concentration. The points represent the mean ± S.D, *p* = 0.539(*n* = 37)

The results are expressed as ng/ml and represent changes to plasma PCT following FURS (Fig. 4). PCT was measured as means of a biomarker for infection. Only a negligible change in PCT concentration between baseline (0.195 ± 0.002 ng/ml) up to 240 min (0.188 ± 0.004 ng/ml) post-FURS was demonstrated. Analysis employing a paired t-test showed no significant change in PCT concentration (*p* = 0.539). No samples analysis was undertaken for PCT at 30 and 120 min post-operatively, due to the high cost of purchasing assay kits and financial restrictions.

### Stone analysis results

As per routine protocol, stone analysis was undertaken at the Department of Pathology, Leicester Royal Infirmary Hospital (UK). Stone composition results are presented in Table 2.

**Table 2:** Stone composition

Main constituent of stone(s)	Patients
Calcium oxalate monohydrate	21
Calcium phosphate stones	7
Cystine stones	3
Calcium hydroxyl phosphate	3
Calcium-oxalate dihydrate stones	3

### Post-FURS complications

**Fig. 5:**
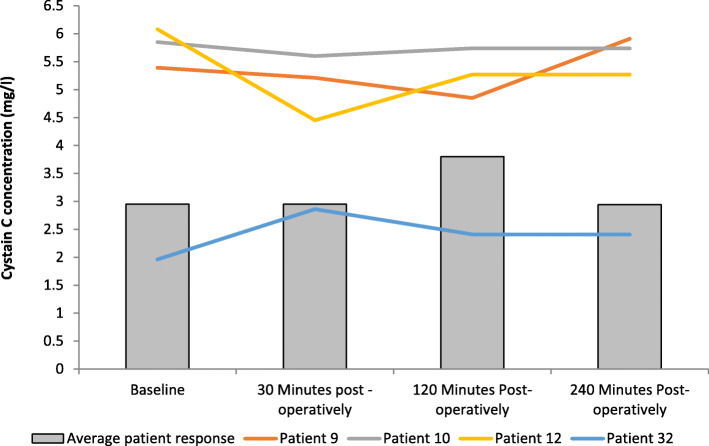
Comparison of Cystatin C concentration across all patients with those developing a complication. Patients 9 (male), 10 (male), and 12 (female) developed urinary tract infection (UTI) with urinary retention. Patient 32 (male) developed urosepsis (Average patient response *n* = 37). Patients 9, 10 and 12 exhibited higher Cystatin C levels than the average group response

**Fig. 6:**
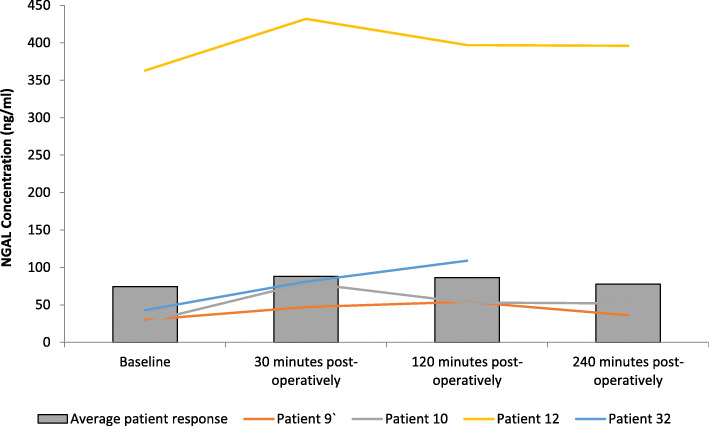
Comparison of NGAL concentration across all patients with those developing a complication. Patients 9 (male), 10 (male), and 12 (female) developed urinary tract infection (UTI) with urinary retention. Patient 32 (male) developed urosepsis (Average patient response *n* = 37). Patients 12 and 32 exhibited higher NGAL levels than the average group response. Missing data is representative of no sample collection or inadequate sample volume for analysis

**Fig. 7:**
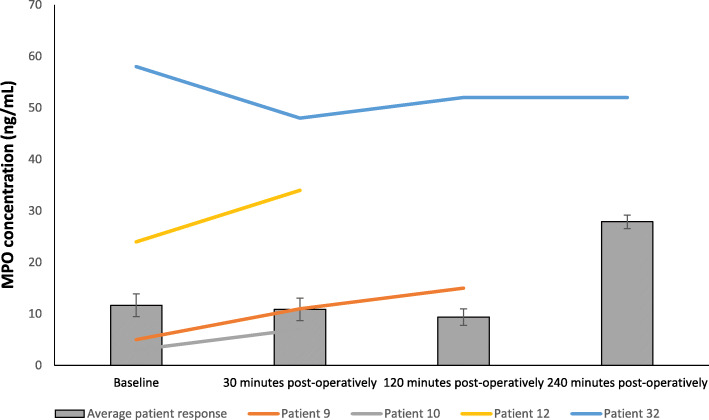
Comparison of MPO concentration across all patients with those developing a complication. Patients 9 (male), 10 (male), and 12 (female) developed urinary tract infection (UTI) with urinary retention. Patient 32 (male) developed urosepsis (Average patient response *n* = 37). Patients 12 and 32 exhibited higher MPO levels than the average group response. Missing data is representative of no sample collection or inadequate sample volume for analysis

**Fig. 8:**
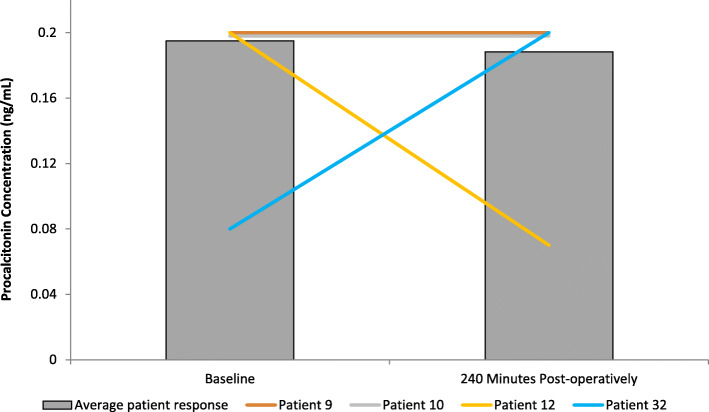
Comparison of PCT concentration across all patients with those developing a complication. Patients 9 (male), 10 (male), and 12 (female) developed urinary tract infection (UTI) with urinary retention. Patient 32 (male) developed urosepsis (Average patient response *n* = 37). Patients 9, 10 and 32 exhibited higher PCT levels than the average group response

One female and two males developed a post-operative urinary tract infection (UTI) with urinary retention. One male patient developed urosepsis. Levels of Cystatin C and NGAL for these patients are compared with the average (*n* = 37) in Figs. 5, 6, 7, 8.

A summary of the main findings from the study are presented in Table 3*.*

**Table 3:** Summary of main findings

➢ A significant increase in NGAL concentration post-FURS for the management of nephrolithiasis (*p* = 0.034).	
➢ No significant differences were seen in Cystatin C, MPO or PCT concentrations post-FURS, for the management of nephrolithiasis (*p* > 0.05)	

## Discussion

The aim of this prospective observational study was to evaluate novel kidney and infection biomarkers, following FURS in the management of nephrolithiasis. Currently, studies reporting on the role of biological markers following FURS are limited.

Serum cystatin C is a non-glycosylated, 13.3-kDa protein belonging to cystatin protease inhibitors [28]. It has shown promise as a replacement for serum creatinine as a potential kidney biomarker [29, 30]. A 2002 meta-analysis based on 46 studies concluded that cystatin C is superior to creatinine for detection of compromised renal function, as well as subsequent renal function monitoring [31]. Although in our study there were no significant changes in Cystatin C levels following FURS, some interesting observations were noted when comparing data of those who developed complications (patients 9, 10, 12 and 32) with those who did not (Fig. 5).

Specifically, patient 9 had developed 3 stones (size 2 mm, 20 and 5 mm; comprising 100% calcium oxalate; duration of procedure 28 min); patient 10 developed 1 stone (size 20 mm; comprising 80% calcium phosphate and 20% calcium oxalate monohydrate; duration of procedure 32 min); and patient 12 developed 2 stones (size 20 and 17 mm; comprising 100% calcium phosphate; duration of procedure 98 min). With regards to patients 9, 10 and 12 none were first time stoners, none received pre-operative stents and all three received a stent post-operative. Interestingly, The Cystatin C levels of patients who developed UTIs and subsequent urinary retention (patient 9, 10, and 12) were higher to that of the normal average patient response (Fig. 5). This biomarker, therefore, shows potential promise and warrants further investigation, as for the patients who developed complications, Cystatin C levels rose sharply within a four-hour time period, thus highlighting its sensitivity and potential role as a marker of monitoring patients post FURS.

Recent research has suggested that NGAL is a useful biomarker for measuring renal function and predicting kidney injury [32, 33]. NGAL measured in the present study demonstrated a significant increase in NGAL levels post FURS (*p* = 0.034), with levels peaking between 30 min and 2 h post treatment. Our results compliment findings from a Dede et al. (2015) findings, in 30 patients, that NGAL increased significantly 2 h post-FURS [34]. Interestingly, the present study also highlighted that patients who developed a complication post-FURS (specifically patients 12 and 32; Fig. 6), reported increased NGAL levels compared to the average patient response. Although not confirmative, these initial pilot data provide some interesting results and may warrant further investigation by recruiting larger cohorts.

With respect MPO, no significant changes were observed in this biomarker following FURS. However, and interesting trend of increased MPO levels were observed and reported in patients who developed a complication post-FURS (specifically, patients 9, 12 and 32; Fig. 7). Although MPO has been reported to be increased in various inflammatory conditions and is considered a risk marker for cardiovascular disease [35, 36], here we provide evidence that MPO may also be considered as a potential biomarker for identifying patients with infectious complications post FURS, although it is appreciated that studies involving larger cohorts will be needed to validate this proposition.

Although PCT is considered a reliable biomarker that displays greater specificity than other proinflammatory markers, such as cytokines in identifying patients with sepsis and aiding in the diagnosis of bacterial infections [37]. The results from our pilot study were disappointing in that PCT levels did not appear to significantly increase in the four patients that developed complications. Meisner et al. (1999) have previously reported on the kinetic profiles of different biomarkers of bacterial infection, illustrating that PCT levels in plasma tend not to peak until 10–12 h following infection [38, 39]. In the present study, blood sampling was not undertaken beyond > 4 h period due to ethical restirctions, which may provide an explination as to why no significant changes to PCT levels were reported. It is postulated that the time frame (and a limitation of the present study) was too short to see elevated PCT in patients with complications [38]. However, in addition, further evidence is provided here, in that that the other biomarkes investigated in the present study (i.e. Cystatin C, NGAL and MPO) may provide more sensitivity for detecting complications ealier than PCT follwoing FURS.

A limiting factor associated with this study was the relatively small number of patients recruited and the number of sampling time points. It would have been beneficial to have obtained further blood samples at time point beyond 4 h (e.g. up to 24–48 h), but as these were day-case patients obtaining additional samples was not possible. In addition, the inclusion of a comparative control group, possibly involving patients undergoing other urological procedures such as shock wave lithotripsy (SWL) or TUR-B without fluid pressure to the controlling system (reflux out ruled) thereby identifying the potential influence of anaesthesia may have been identified. However, restriction to current ethics did not permit the inclusion of such control groups.

In summary, we report that this study provides a basis to undertake further investigations involving biomarkers for monitoring FUR patients during the post-operative period. It is envisaged that when these preliminary studies have been performed and authenticated in larger cohorts, a panel of biomarkers (or biomarker profile) may be produced that can help identify or predict those patients that will develop post-operative complications following FURS.

## Conclusions

Changes seen to biomarkers such as Cystatin C, NGAL and MPO in our observational clinical pilot-study may warrant further investigation, involving larger cohorts, to fully understand the role of these biomarkers and their potential association with post-operative complications which can develop following FURS.

## Data Availability

All data generated or analysed during this study are included in the published article.

## Abbreviations

CRP: C reactive protein
EDTA: Ethylene diaminetetraacetic acid
ELISA: Enzyme-linked Immunoassay
FURS: Flexible ureteroscopy
HOCl: Hypochlorous acid
ICU: Intensive care unit
Iqr: Interquartile range
MPO: Myeloperoxidase
NGAL: Neutrophil gelatinase-associated lipocalin
NO: Nitric Oxide
PCT: Procalcitonin
SD: Standard deviation
SIRS: Systemic inflammatory response syndrome
SWL: Shock wave lithotripsy
UTI: Urinary tract infection
